# Traditional Chinese medicine for oral squamous cell carcinoma: A Bayesian network meta-analysis protocol

**DOI:** 10.1097/MD.0000000000022955

**Published:** 2020-10-23

**Authors:** Dong Wang, XiaoJie Duan, Yuhui Zhang, Zhen Meng, Jing Wang

**Affiliations:** a Department of Stomatology, Liaocheng People's Hospital; b Medical College of Liaocheng University, Liaocheng, Shandong; c The State Key Laboratory Breeding Base of Basic Science of Stomatology (Hubei-MOST) and Key Laboratory for Oral Biomedical Ministry of Education, School and Hospital of Stomatology, Wuhan University, Wuhan, Hubei; d Key Lab of Precision Biomedicine & Department of Stomatology, Liaocheng People's Hospital; e College of Stomatology, Shandong First Medical University, Liaocheng, Shandong Province, P.R. China.

**Keywords:** Bayesian network meta-analysis, efficacy, oral squamous cell carcinoma, traditional Chinese medicine

## Abstract

**Background:**

Traditional Chinese medicine is frequently used for malignant tumors in China, but in clinical practice, most practitioners choose appropriate Chinese medicines based on personal experience. In our study, Bayesian network meta-analysis will be used to identify differences in efficacy and safety between diverse traditional Chinese drugs for oral squamous cell carcinoma (OSCC).

**Methods:**

Relevant randomized controlled trials and prospective controlled clinical trials were searched from Medline, PubMed, Cochrane Library, Google Scholar, Excerpt Medica Database, Web of Science, China National Knowledge Infrastructure, China Scientific Journal Database, Chinese Biomedical Literature Database, and Wanfang Database from their establishment to September 2020. Study selection and data extraction will be performed independently by 2 researchers. Aggregate Data Drug Information System and R software were used for data synthesis. The evidentiary grade of the results will be also evaluated.

**Results:**

The results of this study will be published in a peer-reviewed journal, and provide reliable evidence for different traditional Chinese drugs on OSCC.

**Conclusions:**

The findings will provide reference for evaluating the efficacy and safety of different traditional Chinese medicine for OSCC, and provide a helpful evidence for clinicians to formulate the best adjuvant treatment strategy for OSCC patients.

**Trial registration number:**

INPLASY202090082.

## 1 Introduction

Oral cancers (OC) are the sixth most common cancer worldwide, accounting for about 2% of all cancers, with an estimated 354,864 new cases worldwide in 2018.^[1,2]^ The incidence of OC varies by geographic area, and the incidence rate is higher in some parts of Europe and South Central Asia.^[3–5]^ There is overwhelming evidence that tobacco use, alcohol consumption and betel quid chewing are the main risk factors in the aetiology of intraoral cancer.^[3,6–11]^ The most common cancer of the OC is the squamous cell carcinoma (over 95% of all OC) that arises from the lining of the OC.^[12–14]^ Currently, only a little more than 50% of these patients will survive beyond 5 years; this rate has remained unchanged despite the advances in oncology treatment.^[13,15]^ The gold standard for treatment is surgery, radiation therapy, or a combination of the 2, depending on the extent of the disease.^[15–21]^ However, their clinical applications are limited by failing to thoroughly eliminate tumor cells, drug resistance and other adverse effects.^[13,15,17]^ In view of these drawbacks to conventional therapy, there is a growing interest in the development of a new regimen with better tolerance and lower toxicity for patients with oral squamous cell carcinoma (OSCC).

**Figure 1:**
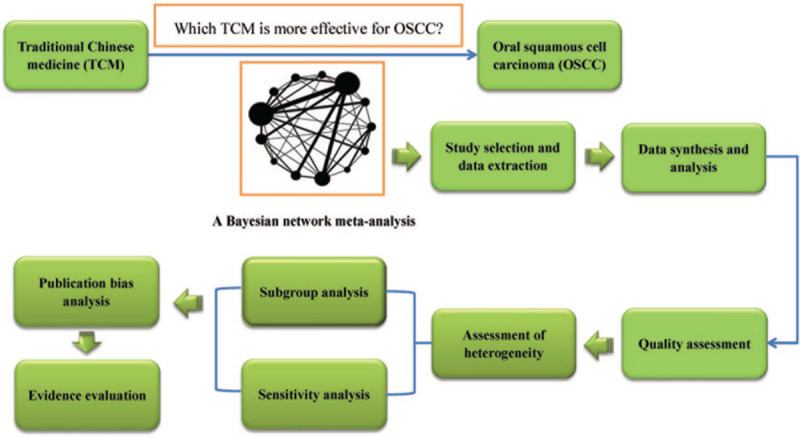
Work flow of the present study.

In light of the limitations of the above treatments, traditional Chinese medicine (TCM), as an essential component of complementary and alternative medicine, has gained more and more attention for malignant tumors.^[22–28]^ TCM are prepared by extracting and purifying the effective and active compounds from herbs, insects or animals via modern scientific techniques and methods. The anticancer TCM are mainly used for adjuvant radiotherapy and chemotherapy against tumors by reducing toxicity, enhancing efficiency, ameliorating symptoms, and improving the immune status in clinical use.^[22–28]^ Although several clinical trials have analyzed the effectiveness of different TCM therapies for OSCC, no comparison of efficacy between different TCM has been made.^[29–31]^ As a result, there is no decision-making conclusion as to which TCM preparation to choose in clinical practice. Therefore, the authors aim to examine the comparative effectiveness of different traditional Chinese preparations for OSCC by conducting a Bayesian network meta-analysis (Fig. 1, Work flow of the present study).

### 1.1 Review question

Which traditional Chinese preparation is more effective for the treatment of patients with OSCC?

### 1.2 Objective

A Bayesian network meta-analysis will be performed to systematically evaluate the comparative effectiveness of different traditional Chinese preparations for OSCC.

## 2 Methods

The protocol of our meta-analysis will be reported according to Preferred Reporting Items for Systematic Review and Meta-Analysis Protocols guidelines.^[32]^ Our protocol has been registered on the International Platform of Registered Systematic Review and Meta-Analysis Protocols (INPLASY). The registration number was INPLASY202090082 (DOI number is 10.37766/inplasy2020.9.0082, https://inplasy.com/inplasy-2020-9-0082/).

### 2.1 Ethics

Given that the meta-analysis is a secondary research which based on some previously published data, ethical approval is not necessary for our research.

### 2.2 Eligibility criteria

#### 2.2.1 Types of studies

Randomized controlled trials (RCTs) and quasi-RCTs or prospective controlled clinical trials that investigated the efficacy and safety of TCM for patients diagnosed with OSCC will be included in this systematic review. There will be no restrictions for blinding, population characteristics and duration of trials.

#### 2.2.2 Type of participants

Patients with histologically proved squamous cell carcinomas of the oral cavity were included in this study. No restrictions regarding age, gender, racial, region, education, and economic status. Patients with other malignancies are not included.

#### 2.2.3 Types of interventions

In the experimental group, OSCC patients must be treated with TCM alone or in combination with other pharmacological interventions. TCM involving extracts from herbs or insects or animals, single or mixture formulas regardless of their compositions or forms. There will be no restrictions with respect to dosage, duration, frequency, or follow-up time of treatment.

#### 2.2.4 Comparator

There will be no restrictions with respect to the type of comparator. The comparators are likely to include placebo, western medical therapies, supportive care, and other therapeutic methods.

#### 2.2.5 Type of outcome measurements

##### 2.2.5.1 Primary outcomes

- Overall response rate and disease control rate;
- Overall survival, the time from the date of randomization to death from any cause.

##### 2.2.5.2 Secondary outcomes

- Quality of life obtained from the corresponding scale.
- Immune function indicators: CD3^+^, CD4^+^, CD8^+^, NK cells percentage, CD4^+^/CD8^+^ cell ratios, and serum cytokines level (IL-2, IL-4, IFN-γ and TNF-α);
- Adverse effects: treatment-related toxicity was graded from 0 to IV according to the World Health Organization recommendations.

#### 2.2.6 Exclusion criteria

Duplicated studies, papers without sufficient available data, non-comparative clinical trials, case reports and series, meta-analysis, literature reviews, meeting abstracts, and other unrelated studies will be excluded from analysis.

### 2.3 Information sources

Electronic databases including relevant RCTs, quasi-RCTs and high-quality prospective cohort studies were searched from Medline, PubMed, Cochrane Library, Google Scholar, Excerpt Medica Database, Web of Science, China National Knowledge Infrastructure, China Scientific Journal Database, Chinese Biomedical Literature Database, and Wanfang Database will be systematically searched for eligible studies from their inception to September 2020. Language is limited with English and Chinese.

### 2.4 Search strategy

To perform a comprehensive and focused search, experienced systematic review investigators will be invited to develop a search strategy. The plan searched terms are as follows: “oral cancer” or “oral cavity cancer” or “mouth cancer” or “oral cavity carcinomas” or “oral squamous cell carcinoma” or “kou qiang ai” or “kou qiang lin zhuang xi bao ai” or “OC” or “OCC” or “OSCC” and “traditional Chinese medicine” or “traditional Chinese drug” or “Chinese herbal preparation” or “traditional Chinese preparation” or “Chinese materia medica preparation” or Chinese patent medicine” or “zhongyao” or “TCM” or “TCD” et al. The preliminary retrieval strategy for PubMed is provided in Table 1, which will be adjusted in accordance with specific databases.

**Table 1:**
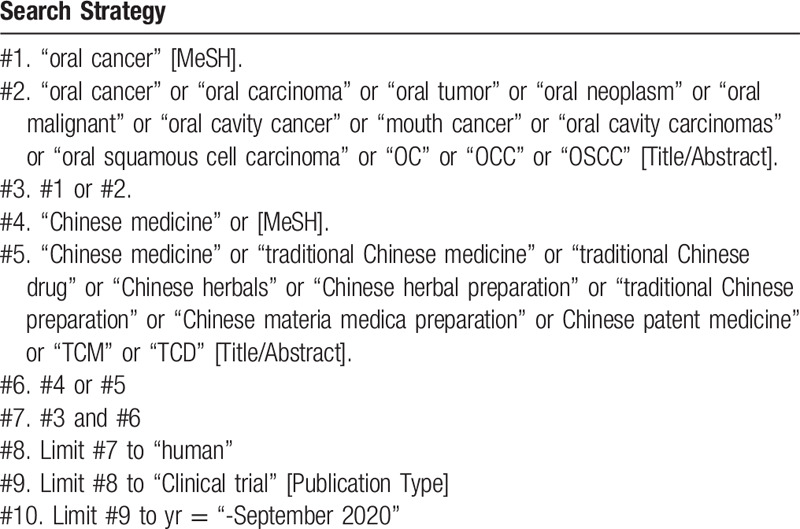
Searching strategy in PubMed.

### 2.5 Study selection and management

**Figure 2:**
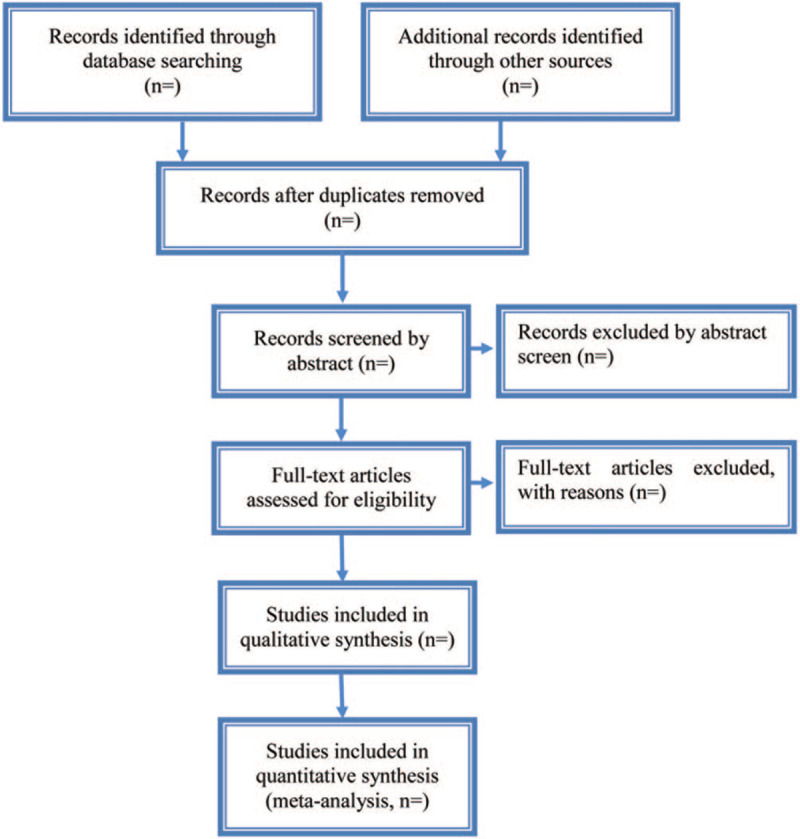
Study selection process for the meta-analysis.

We will use a 2-step process to assess the results of the literature search. First, all qualified documents will be extracted in the form of title and abstract, and preliminary screening will be conducted based on this information. On the basis of the previous step, the full text of the qualified literature will be obtained and further screened. All screening processes will be performed independently by the 2 authors (Dong Wang and XiaoJie Duan), and the reasons for each rejection will be documented. Disagreements between the 2 reviewers will be resolved by discussing with the third investigator (Yuhui Zhang). A Preferred Reporting Items for Systematic Review and Meta-Analysis-compliant flow chart (Fig. 2) will be used to describe the selection process of eligible literatures. Endnote X7 software will be used for literature managing and records searching.

### 2.6 Data extraction and management

After screening the literature, the 2 authors (Dong Wang and XiaoJie Duan) will independently extract the information contained in the eligible literature to form a document feature table.

The extracted data are as follows:

- Study characteristics and methodology: country of study, the first author's name, year of publication, randomization, sample size, periods of data collection, follow-up duration, outcome measures, inclusion and exclusion criteria, et al.
- Participant characteristics: age, gender, tumor stage (staging of the tumor according to the staging system of the International Union Against Cancer-IUAC), tumor size, diagnostic criteria, et al.
- Interventions: therapeutic means, dose, administration route, course of treatment, and duration of treatment, et al.
- Outcome and other data: overall response rate, disease control rate, Overall survival, quality of life, immune indexes [(CD3^+^, CD4^+^, CD8^+^, NK cells percentage, and CD4^+^/CD8^+^ cell ratios, and serum cytokines level (IL-2, IL-4, IFN-γ, and TNF-α)], and adverse effects, et al.

### 2.7 Quality assessment

Two review authors (Dong Wang and XiaoJie Duan) will independently assess the quality of the included RCTs. The assessment tool is provided by Cochrane, which includes 7 items: random sequence generation, allocation concealment, blinding of participants and personnel, blinding of outcome assessment, incomplete outcome data, selective reporting and other bias.^[33,34]^ Each item will be evaluated at 3 levels: low risk, unclear, and high risk. Effective Practice and Organisation of Care guidelines will be used to assess the risks of non-RCTs.^[35]^ Any disagreements will be resolved via discussion with a third researcher (Yuhui Zhang).

### 2.8 Data synthesis

First, we will conduct a conventional pairwise meta-analysis of the direct comparison results obtained from the literature. Continuous data will be presented as mean difference or standardized mean difference with their confidence intervals (CIs). Dichotomous data will be recorded as odds ratio with 95% CIs. Second, for the results of indirect comparison, the authors will use aggregate data drug information system and R software to conduct network meta-analysis based on random effect model.^[36,37]^ We will calculate the pooled estimates and 95% CIs of the mean difference/standardized mean difference and odds ratio for primary outcomes. To present indirect comparisons of traditional Chinese drugs, we will make a network diagram. The network graph is mainly composed of nodes and lines. Among them, the node represents a kind of therapy, and the nodes connected by lines indicate that there is a direct or indirect comparative relationship between the 2.^[36]^ The node size represents the number of subjects receiving this therapy.^[36]^ The thickness of the line represents the number of studies.^[36]^ Then, we will analyze the outcomes from all direct or indirect comparisons to assess which traditional Chinese drug for OSCC is most effective and estimate the rank probabilities of all the groups based on the Markov chain Monte Carlo method.

### 2.9 Assessment of heterogeneity

Heterogeneity of treatment effects across trials was assessed by *χ*^2^ statistics and the *I*^2^ statistics.^[38]^ When the *P*-value was >.1, and *I*^2^ was <50%, it suggested that there was no statistical heterogeneity and the Mantel–Haenszel fixed-effects model was used for meta-analysis. Otherwise, a random-effects mode will be used to calculate the outcomes.

### 2.10 Subgroup and meta-regression analysis

When the *P*-value was <.1, and *I*^2^ was >50%, we explored sources of heterogeneity with respect to age, tumor stage, region and types of TCM by subgroup analysis and meta-regression.

### 2.11 Sensitivity analysis

Sensitivity analysis will be conducted to assess the reliability and robustness of the aggregation results via eliminating trials with low-quality. A summary table will report the results of the sensitivity analyses.

### 2.12 Publication bias

Funnel plot will be performed to analyze the existence of publication bias if 10 or more studies are included in this meta-analysis. If the funnel chart has poor symmetry, it indicates publication bias.^[39]^

### 2.13 Assess the quality of evidence

The evidence grade will be assessed by using the guidelines of the Grading of Recommendations, Assessment, Development, and Evaluation. The quality of all evidence will be assessed at 4 levels: high, moderate, low, and very low.^[40]^

## 3 Discussion

Multiple studies have recognized that TCM have a unique advantage in the treatment of malignant tumors by inhibiting the growth of cancer cells, enhancing immunity of human body, decreasing cancer relapses and metastases, and mitigating the progress of the disease.^[22–31]^ In spite of a growing number of studies on TCM for patients with malignant tumors in the late years,^[29–31]^ there is rare evidence to validate the difference in efficacy and safety among various TCM drugs for OSCC. Considering that high-quality meta-analysis could provide reliable guidance for clinicians, the authors intend to complete a network meta-analysis based on Bayesian model. Through direct or indirect comparison, the author plans to rank the efficacy and safety of difference TCM drugs for OSCC. We hope that the study results will help to figure out which one or which combination of these interventions has the relatively optimal effect and safety and provide decision-making reference for clinicians, patients, and policy-makers to a certain extent.

There are some limitations that may affect the drawn conclusion. There may be a language bias with the limitation of English and Chinese studies. In addition, due to the different TCM drugs, tumor stage and duration of treatment among included trials, that may cause a certain degree of heterogeneity.

## Author contributions

**Conceptualization:** Dong Wang, Jing Wang.

**Data curation:** Dong Wang, XiaoJie Duan, Yuhui Zhang.

**Formal analysis:** Dong Wang, XiaoJie Duan, Yuhui Zhang.

**Funding acquisition:** Zhen Meng.

**Investigation:** Dong Wang, XiaoJie Duan, Yuhui Zhang.

**Methodology:** Dong Wang, XiaoJie Duan, Yuhui Zhang, Zhen Meng.

**Project administration:** Jing Wang.

**Resources:** Dong Wang, Jing Wang.

**Software:** Dong Wang, Jing Wang.

**Supervision:** Dong Wang, Jing Wang.

**Validation:** Zhen Meng, Jing Wang.

**Visualization:** Dong Wang, XiaoJie Duan, Yuhui Zhang.

**Writing – original draft:** Dong Wang, XiaoJie Duan, Yuhui Zhang.

**Writing – review & editing:** Zhen Meng, Jing Wang.

## References

[1] Bray F Ferlay J Soerjomataram I Global cancer statistics 2018: GLOBOCAN estimates of incidence and mortality worldwide for 36 cancers in 185 countries. CA Cancer J Clin 2018;68:394–424. 30207593 10.3322/caac.21492

[2] Ferlay J Colombet M Soerjomataram I Estimating the global cancer incidence and mortality in 2018: GLOBOCAN sources and methods. Int J Cancer 2019;144:1941–53. 30350310 10.1002/ijc.31937

[3] Chan KK Glenny AM Weldon JC Interventions for the treatment of oral and oropharyngeal cancers: targeted therapy and immunotherapy. Cochrane Database Syst Rev 2015;12:CD010341. 10.1002/14651858.CD010341.pub2 PMC9465394 26625332

[4] Parkin DM Bray F Ferlay J Global cancer statistics, 2002. CA Cancer J Clin 2005;55:74–108. 15761078 10.3322/canjclin.55.2.74

[5] Montero PH Patel SG Cancer of the oral cavity. Surg Oncol Clin N Am 2015;24:491–508. 25979396 10.1016/j.soc.2015.03.006 PMC5018209

[6] La Vecchia C Tavani A Franceschi S Epidemiology and prevention of oral cancer. Oral Oncol 1997;33:302–12. 9415327 10.1016/s1368-8375(97)00029-8

[7] Macfarlane GJ Zheng T Marshall JR Alcohol, tobacco, diet and the risk of oral cancer: a pooled analysis of three case-control studies. Eur J Cancer B Oral Oncol 1995;31B:181–7. 7549758 10.1016/0964-1955(95)00005-3

[8] Ernani V Saba NF Oral cavity cancer: risk factors, pathology, and management. Oncology 2015;89:187–95. 26088938 10.1159/000398801

[9] Ghantous Y Abu Elnaaj I Global incidence and risk factors of oral cancer. Harefuah 2017;156:645–9. 29072384

[10] Kadashetti V Chaudhary M Patil S Analysis of various risk factors affecting potentially malignant disorders and oral cancer patients of Central India. J Cancer Res Ther 2015;11:280–6. 26148585 10.4103/0973-1482.151417

[11] Kumar M Nanavati R Modi TG Oral cancer: etiology and risk factors: a review. J Cancer Res Ther 2016;12:458–63. 27461593 10.4103/0973-1482.186696

[12] Andreadis C Vahtsevanos K Sidiras T 5-Fluorouracil and cisplatin in the treatment of advanced oral cancer. Oral Oncol 2003;39:380–5. 12676258 10.1016/s1368-8375(02)00141-0

[13] Cerrati EW Nguyen SA Farrar JD The efficacy of photodynamic therapy in the treatment of oral squamous cell carcinoma: a meta-analysis. Ear Nose Throat J 2015;94:72–9. 25651350 10.1177/014556131509400208

[14] van Dijk BA Brands MT Geurts SM Trends in oral cavity cancer incidence, mortality, survival and treatment in the Netherlands. Int J Cancer 2016;139:574–83. 27038013 10.1002/ijc.30107

[15] Day TA Davis BK Gillespie MB Oral cancer treatment. Curr Treat Options Oncol 2003;4:27–41. 12525277 10.1007/s11864-003-0029-4

[16] Konkimalla VB Suhas VL Chandra NR Diagnosis and therapy of oral squamous cell carcinoma. Expert Rev Anticancer Ther 2007;7:317–29. 17338652 10.1586/14737140.7.3.317

[17] Fridman E Na’ara S Agarwal J The role of adjuvant treatment in early-stage oral cavity squamous cell carcinoma: an international collaborative study. Cancer 2018;124:2948–55. 29757457 10.1002/cncr.31531 PMC6607430

[18] Inagi K Takahashi H Okamoto M Treatment effects in patients with squamous cell carcinoma of the oral cavity. Acta Otolaryngol Suppl 2002;547:25–9. 10.1080/000164802760057527 12212589

[19] Omura K Current status of oral cancer treatment strategies: surgical treatments for oral squamous cell carcinoma. Int J Clin Oncol 2014;19:423–30. 24682763 10.1007/s10147-014-0689-z

[20] Gharat SA Momin M Bhavsar C Oral squamous cell carcinoma: current treatment strategies and nanotechnology-based approaches for prevention and therapy. Crit Rev Ther Drug Carrier Syst 2016;33:363–400. 27910740 10.1615/CritRevTherDrugCarrierSyst.2016016272

[21] Hartner L Chemotherapy for oral cancer. Dent Clin North Am 2018;62:87–97. 29126496 10.1016/j.cden.2017.08.006

[22] Mao CG Tao ZZ Wan LJ The efficacy of traditional Chinese Medicine as an adjunctive therapy in nasopharyngeal carcinoma: a systematic review and meta-analysis. J BUON 2014;19:540–8. 24965419

[23] Song YC Hung KF Liang KL Adjunctive Chinese herbal medicine therapy for nasopharyngeal carcinoma: clinical evidence and experimental validation. Head Neck 2019;41:2860–72. 30985039 10.1002/hed.25766

[24] Wang Q Liu H Qiao N Effects of traditional Chinese medicine on salivary glands in the patients with head and neck cancer during radiotherapy. Chin J Integ Trad West Med 1998;18:662–4. 11477859

[25] Wei R Yang DY Jiang WZ Efficacy of Yanshu injection (a compound Chinese traditional medicine) combined with concurrent radiochemotherapy in patients with stage III nasopharyngeal carcinoma. Chin J Oncol 2011;33:391–4. 21875474

[26] Wu T Cui H Xu Y The effect of tubeimoside-1 on the proliferation, metastasis and apoptosis of oral squamous cell carcinoma in vitro. Onco Targets Ther 2018;11:3989–4000. 30022842 10.2147/OTT.S164503 PMC6044352

[27] Yin T Yang G Ma Y Developing an activity and absorption-based quality control platform for Chinese traditional medicine: application to Zeng-Sheng-Ping (antitumor B). J Ethnopharmacol 2015;172:195–201. 26099633 10.1016/j.jep.2015.06.019 PMC4541799

[28] Yang JS Wu CC Lee HZ Suppression of the TNF-alpha level is mediated by Gan-Lu-Yin (traditional Chinese medicine) in human oral cancer cells through the NF-kappa B, AKT, and ERK-dependent pathways. Environ Toxicol 2016;31:1196–205. 25721693 10.1002/tox.22127

[29] Cai XQ Chang XY Guo LL Effect of anti-cancer soup combined with chemotherapy on the immune function and prognosis of patients with advanced oral cavity carcinomas after surgery. Oncol Prog 2018;16:1028–31.

[30] Song YH Song ST Xue LS Observe the effects of Chinese herb complex decocfion on tumour combination chemtherapy on advanced mouth squamous cell carcinoma. Cancer Res Prevent Treat 2002;29:337–8.

[31] Liu XG Geng JH Guan ZM Effect of chemotherapy combined with Astragalus membranaceus on tumor markers of oral cancer. Guangming J Chin Med 2007;22:46–7.

[32] Shamseer L Moher D Clarke M Preferred reporting items for systematic review and meta-analysis protocols (PRISMA-P) 2015: elaboration and explanation. BMJ 2015;350:g7647. 25555855 10.1136/bmj.g7647

[33] Higgins JP Altman DG Gotzsche PC The Cochrane Collaboration's tool for assessing risk of bias in randomised trials. BMJ 2011;343:d5928. 22008217 10.1136/bmj.d5928 PMC3196245

[34] Zeng X Zhang Y Kwong JS The methodological quality assessment tools for preclinical and clinical studies, systematic review and meta-analysis, and clinical practice guideline: a systematic review. J Evid Based Med 2015;8:2–10. 25594108 10.1111/jebm.12141

[35] Grimshaw J McAuley LM Bero LA Systematic reviews of the effectiveness of quality improvement strategies and programmes. Qual Saf Health Care 2003;12:298–303. 12897365 10.1136/qhc.12.4.298 PMC1743751

[36] Li F Xu B Wang P Traditional Chinese medicine non-pharmaceutical therapies for chronic adult insomnia: a Bayesian network meta-analysis protocol. Medicine 2019;98:e17754. 31725615 10.1097/MD.0000000000017754 PMC6867756

[37] van Valkenhoef G Tervonen T Zwinkels T ADDIS: a decision support system for evidence-based medicine. Decis Support Syst 2013;55:459–75.

[38] Jackson D White IR Riley RD Quantifying the impact of between-study heterogeneity in multivariate meta-analyses. Stat Med 2012;31:3805–20. 22763950 10.1002/sim.5453 PMC3546377

[39] Lin L Chu H Quantifying publication bias in meta-analysis. Biometrics 2018;74:785–94. 29141096 10.1111/biom.12817 PMC5953768

[40] Guyatt GH Oxman AD Vist GE GRADE: an emerging consensus on rating quality of evidence and strength of recommendations. BMJ 2008;336:924–6. 18436948 10.1136/bmj.39489.470347.AD PMC2335261

